# Supplementary material for: Single-cell transcriptomic evidence for dense intracortical neuropeptide networks
Source: eLife. 2019 Nov 11;8:e47889. doi: 10.7554/eLife.47889 (PMC6881117; doi:10.7554/eLife.47889)
Supplement: Transparent reporting form [file elife-47889-transrepform.docx]

***eLife’s* transparent reporting form**

We encourage authors to provide detailed information *within their submission* to facilitate the interpretation and replication of experiments. Authors can upload supporting documentation to indicate the use of appropriate reporting guidelines for health-related research (see [EQUATOR Network](http://www.equator-network.org/%20)), life science research (see the [BioSharing Information Resource](https://biosharing.org/)), or the [ARRIVE guidelines](http://www.plosbiology.org/article/info:doi/10.1371/journal.pbio.1000412) for reporting work involving animal research. Where applicable, authors should refer to any relevant reporting standards documents in this form.

If you have any questions, please consult our Journal Policies and/or contact us: [editorial@elifesciences.org](mailto:editorial@elifesciences.org).

**Sample-size estimation**

- You should state whether an appropriate sample size was computed when the study was being designed
- You should state the statistical method of sample size computation and any required assumptions
- If no explicit power analysis was used, you should describe how you decided what sample (replicate) size (number) to use

Please outline where this information can be found within the submission (e.g., sections or figure legends), or explain why this information doesn’t apply to your submission:

Our study is an analysis of a large transcriptomic dataset that is now freely available for download in its entirety and is described fully in a rigorously peer-reviewed publication (Tasic, et al., Nature 563:72-78, 2018). That publication is cited extensively throughout the present report and discusses details of sample size and statistical power extensively.

**Replicates**

- You should report how often each experiment was performed
- You should include a definition of biological versus technical replication
- The data obtained should be provided and sufficient information should be provided to indicate the number of independent biological and/or technical replicates
- If you encountered any outliers, you should describe how these were handled
- Criteria for exclusion/inclusion of data should be clearly stated
- High-throughput sequence data should be uploaded before submission, with a private link for reviewers provided (these are available from both GEO and ArrayExpress)

Please outline where this information can be found within the submission (e.g., sections or figure legends), or explain why this information doesn’t apply to your submission:

All details of biological and technical replication, and all criteria for exclusion of data are described at length in the Tasic 2018 resource publication which we cite extensively throughout the present work.

**Statistical reporting**

- Statistical analysis methods should be described and justified
- Raw data should be presented in figures whenever informative to do so (typically when N per group is less than 10)
- For each experiment, you should identify the statistical tests used, exact values of N, definitions of center, methods of multiple test correction, and dispersion and precision measures (e.g., mean, median, SD, SEM, confidence intervals; and, for the major substantive results, a measure of effect size (e.g., Pearson's r, Cohen's d)
- Report exact p-values wherever possible alongside the summary statistics and 95% confidence intervals. These should be reported for all key questions and not only when the p-value is less than 0.05.

Please outline where this information can be found within the submission (e.g., sections or figure legends), or explain why this information doesn’t apply to your submission:

Cell type clustering and statistical analyses are given and justified in Tasic et al 2018. Code and supplementary data files have been placed in a GitHub online repository which is cited in the first paragraph of our Results section. Additional details of statistical analysis of the results presented in Fig. 4 are compiled in a Supplementary Methods section linked to the legend of Fig. 4 and included in our main text file. An R script program Neuropeptides.R executes all the analyses in the figures except for Fig. 4 which is in Python. All data and library dependencies are cited in the code files which will be found in the linked GitHub repository.

(For large datasets, or papers with a very large number of statistical tests, you may upload a single table file with tests, Ns, etc., with reference to sections in the manuscript.)

**Group allocation**

- Indicate how samples were allocated into experimental groups (in the case of clinical studies, please specify allocation to treatment method); if randomization was used, please also state if restricted randomization was applied
- Indicate if masking was used during group allocation, data collection and/or data analysis

Please outline where this information can be found within the submission (e.g., sections or figure legends), or explain why this information doesn’t apply to your submission:

The only group allocation matter at possible issue here is the division of the Tasic et al. 2018 dataset into two groups (VISp and ALM cortex), which was established on straightforward anatomical grounds by highly experienced anatomists, as described in the Tasic, et al. 2018 publication as cited throughout the present work.

**Additional data files (“source data”)**

- We encourage you to upload relevant additional data files, such as numerical data that are represented as a graph in a figure, or as a summary table
- Where provided, these should be in the most useful format, and they can be uploaded as “Source data” files linked to a main figure or table
- Include model definition files including the full list of parameters used
- Include code used for data analysis (e.g., R, MatLab)
- Avoid stating that data files are “available upon request”

Please indicate the figures or tables for which source data files have been provided:

Intermediate data products and code for generating Tables 1-3 and for Figures 1-6 are deposited in the GitHub repository linked from the first paragraph of our Results section. Figure 7 is a purely schematic summary drawing and involves no actual source data or code.
